# Supplementary material for: The F-Actin-Binding MPRIP Forms Phase-Separated Condensates and Associates with PI(4,5)P2 and Active RNA Polymerase II in the Cell Nucleus
Source: Cells. 2021 Apr 8;10(4):848. doi: 10.3390/cells10040848 (PMC8068864; doi:10.3390/cells10040848)
Supplement: Supplementary file 1 [file cells-10-00848-s001.zip › Supplementary/Supplementary Information.docx]

Supplementary Information

The F-Actin-Binding MPRIP Forms Phase-Separated
Condensates and Associates with PI(4,5)P2 and Active RNA Polymerase II in the Cell Nucleus

****Can Balaban****^1^****, Martin Sztacho****^1^****, Michaela Blažíková**** ^2^ ****and Pavel Hozák**** ^1,2, *^

^1^ Department of Biology of the Cell Nucleus, Institute of Molecular Genetics of the Czech Academy of Sciences, v.v.i., 142 20, Prague, Czech Republic

^2^ Microscopy Centre, Institute of Molecular Genetics of the Czech Academy of Sciences, v.v.i., 142 20, Prague, Czech Republic

***** Correspondence: hozak@img.cas.cz;

Received: date; Accepted: date; Published: date


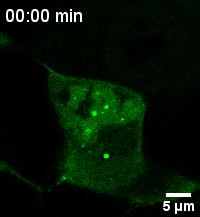


**Video S1: Live cell imaging of a cell overexpressing GFP-MPRIP. The video was recorded approximately for 8 hours, imaging one frame per 5 minutes (for details, see MM). The cell shows many GFP-MPRIP condensates in nucleus and a few in cytoplasm. These condensates change their shape to fibers at the 4th hour of the video, which corresponds to 14th hour after transfection. Bar shows 5 µm. The frame rate of the video is 5 frame per second.**


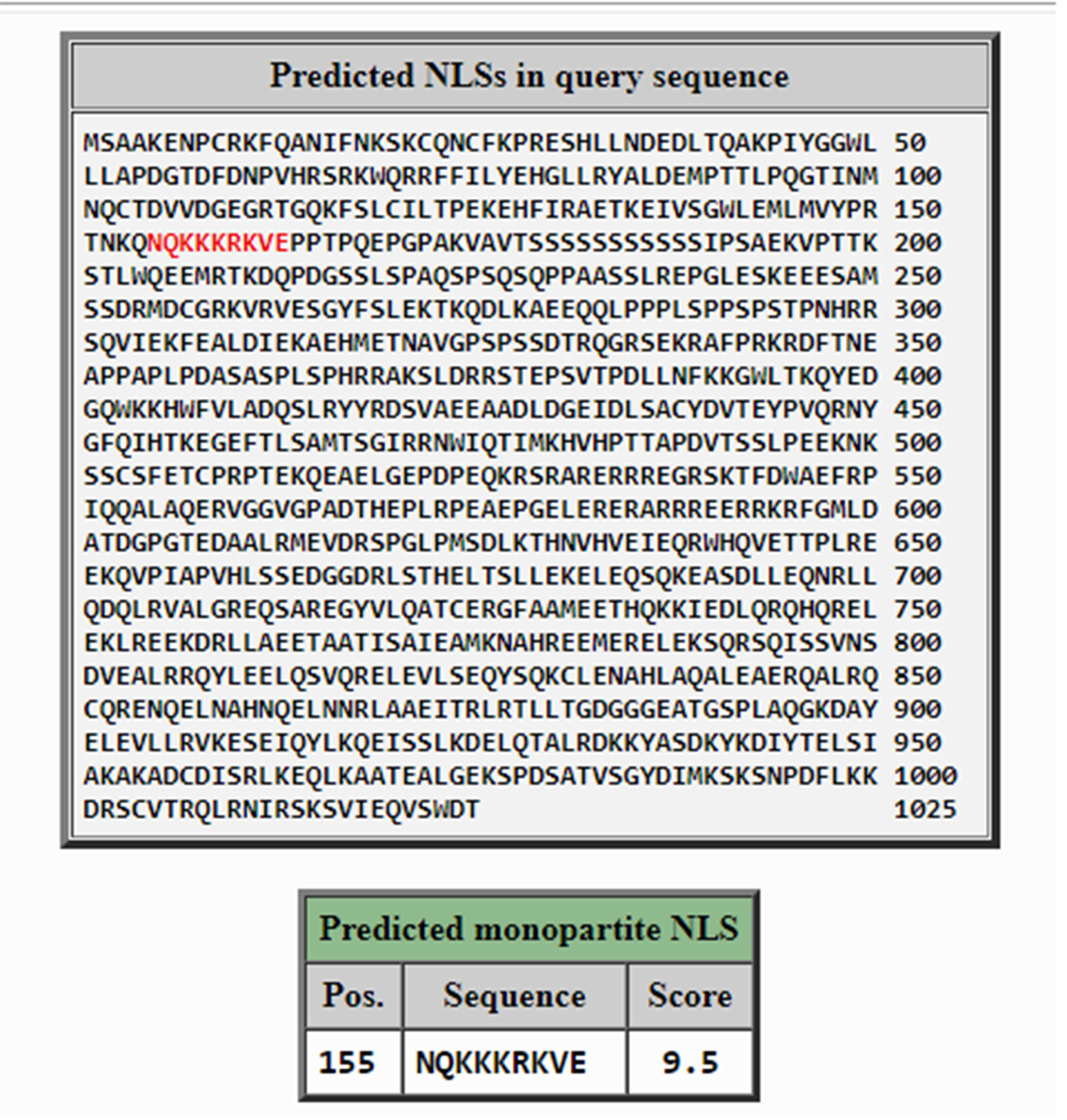


**Figure S1: Predicted NLS sequence of MPRIP protein. The cNLS Mapper prediction tool shows a monopartite NLS sequence covering amino acids in position 155 to 164. The strength of the prediction is: 9.5/10.**


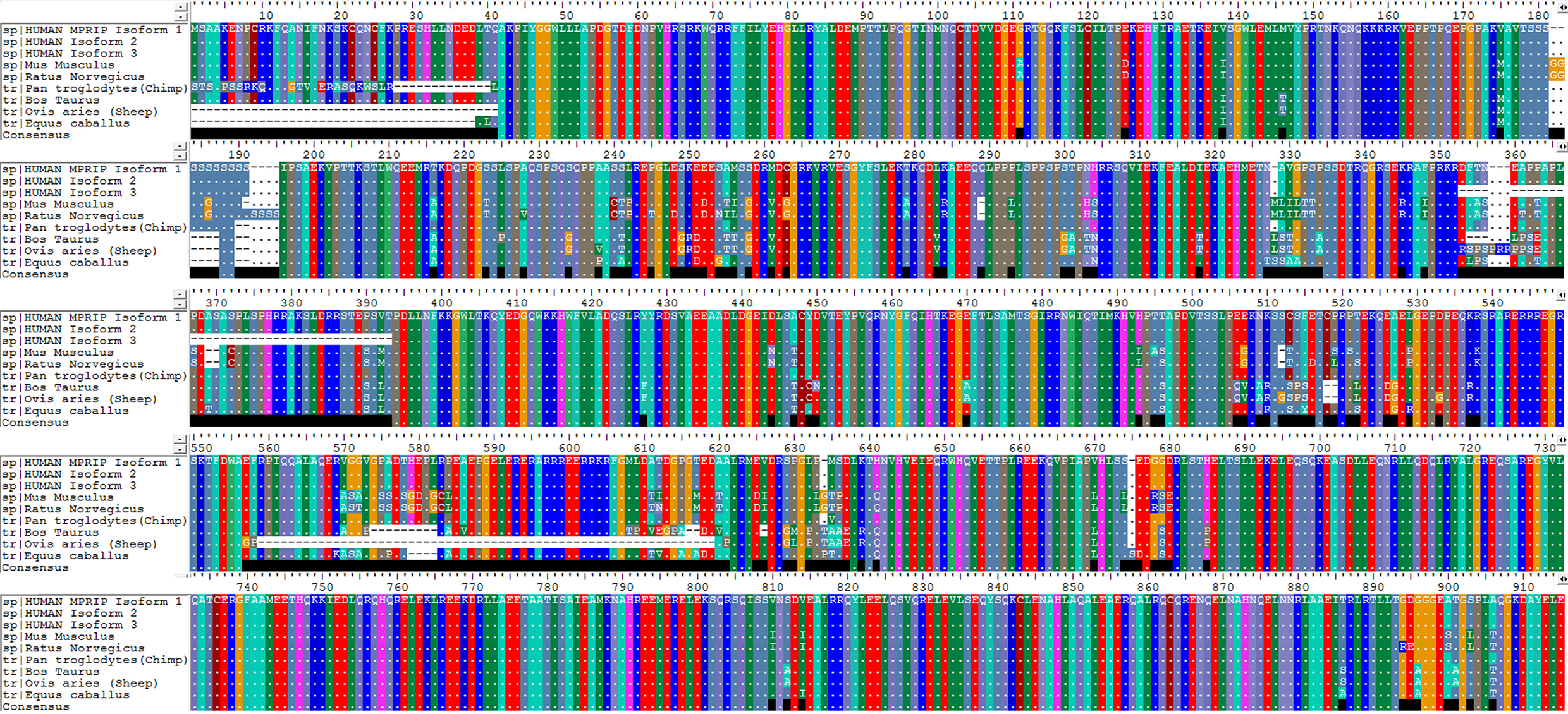


**Figure S2: Clustal Omega test on eight mammalian MPRIP sequences. The first three sequences are the human isofroms of MPRIP, followed by orthologues from Mus Musculus (House mouse), Ratus Norvegicus (Brown Rat), Pan troglodytes (Chimpanzee), Bos Taurus (Bovine), Ovis aries (Sheep) and Equus caballus (Horse). The last sequence is the consensus generated by pairwise sequence alignment provided by EMBL-EBI servers. Each amino acid is color coded where identical amino acids are shown as “.” and missing amino acid in a sequence were indicated with a “- “. Missing or different amino acid in any of the eight mammalian sequences result a dark gap in the consensus sequence.**


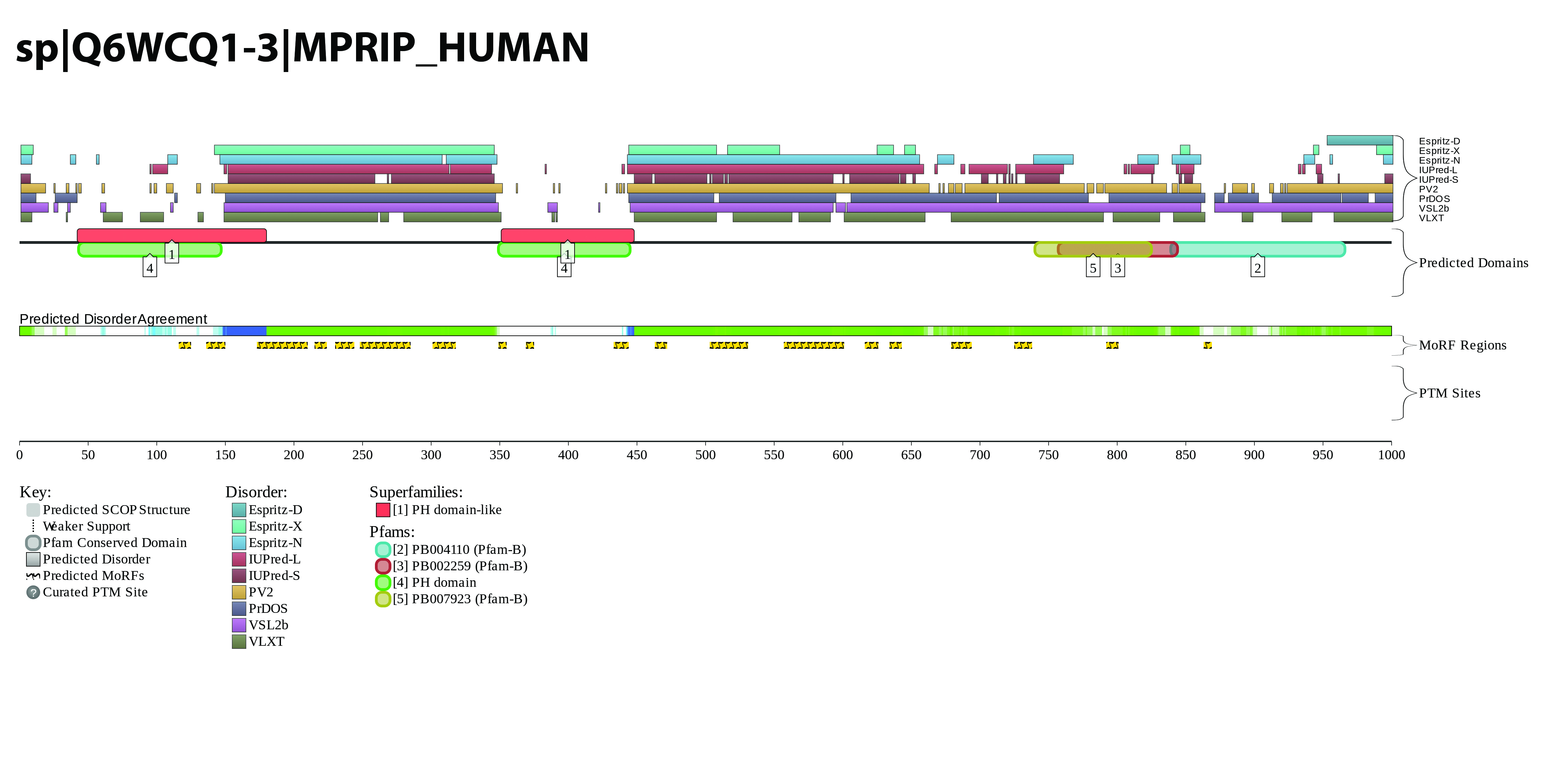


**Figure S3: Structure Determination and IDR of MPRIP Human Isoform 3. Above, the schematic presentation of MPRIP shows the two PH domains and three coiled-coil (Pfam B) domains. The IDR prediction is generated by D2P2 database of disordered protein predictions that run a battery of disorder predictors: VL-XT, VSL2b, PrDOS, PV2, Espritz and IUPred.**

**
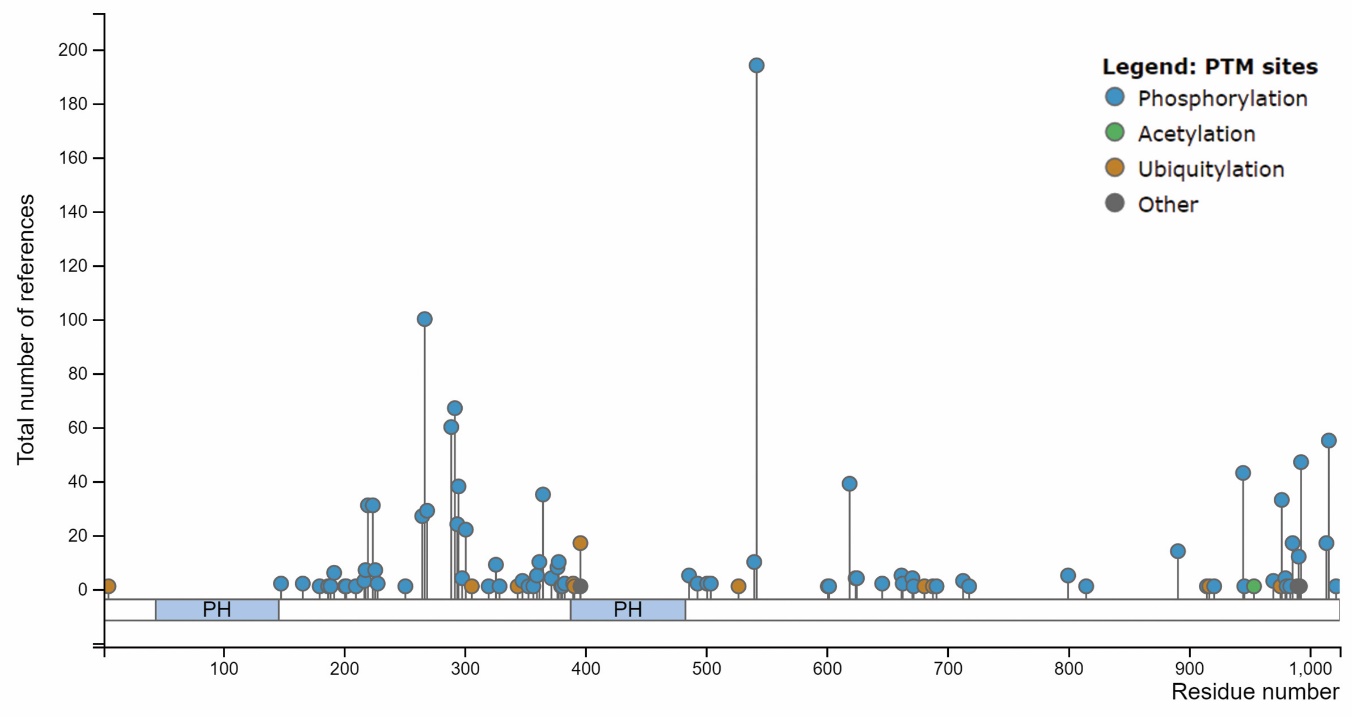
Figure S4: Post-translational modification sites of MPRIP Human Isoform 3. Image obtained from PhosphoSitePlus® shows the amino acid residues which are determined to be post-translationally modified by High Throughput papers. The length of the lollipop plot indicates the number of references published for that residue.**


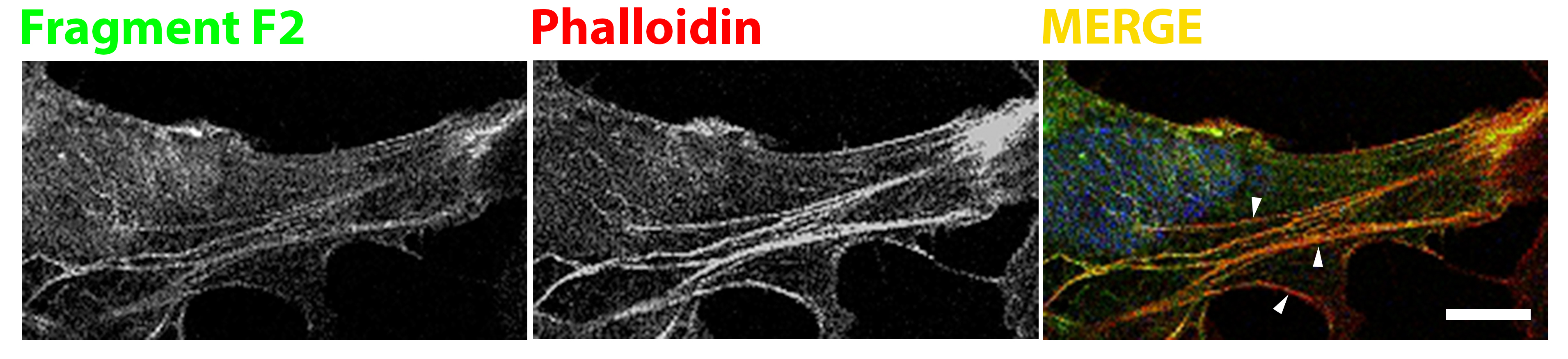


**Figure S5: Fragment F2 of MPRIP localize to actin stress fibers.** Confocal microscopy images show the cellular localization of the overexpressed fragment F2. Arrow heads point to the stress fibers and cortical actin Blue is DAPI, green is GFP-MPRIP and red is the phalloidin staining. Scale bar represent 10 µm.


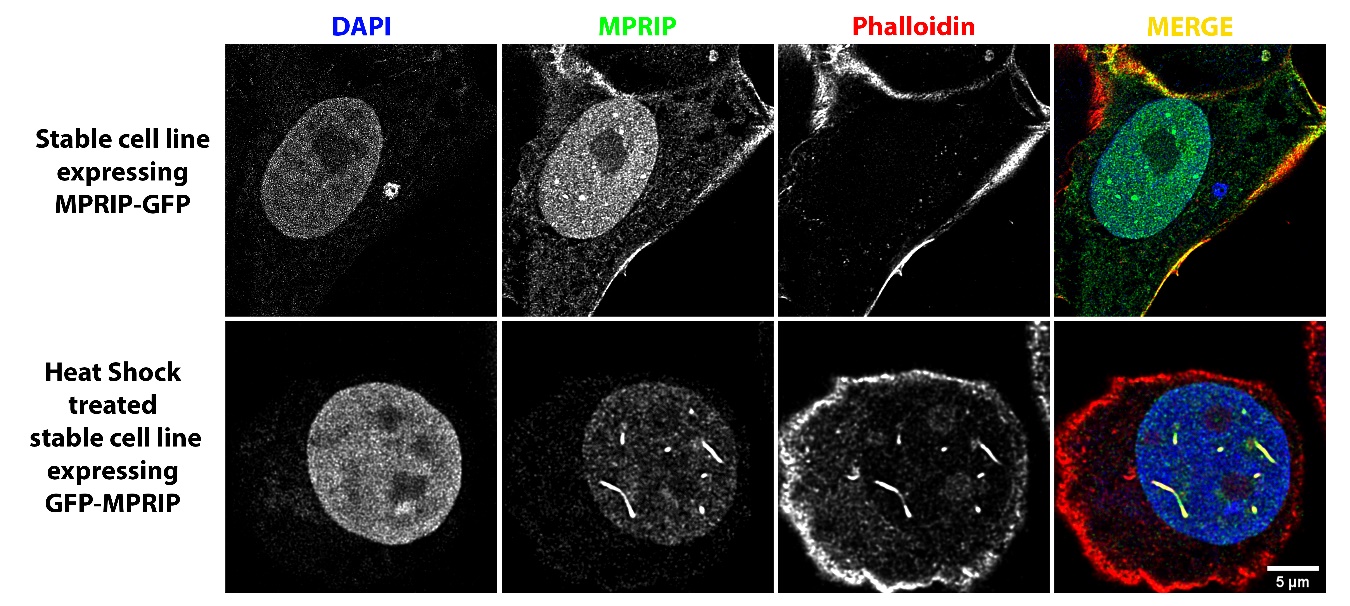


**Figure S6:** **Heat Shock treatment of the stable cell line.** Immunofluorescence experiment on the stable cell line expressing GFP-MPRIP. First row shows untreated cell as a control for heat shock. The cell at the second row is subjected to heat shock for 1 hour at 41°C (for more details, see Methods). Due to the high fluorescence of the fibers, the 488 laser intensity lowered in the heat shock imaging and therefore, DAPI staining looks more intense in the merge image. DAPI in blue, MPRIP in green, Phalloidin in red. Scale bar corresponds to 5 µm. Reproduced three times.
